# Gaze Duration Biases for Colours in Combination with Dissonant and Consonant Sounds: A Comparative Eye-Tracking Study with Orangutans

**DOI:** 10.1371/journal.pone.0139894

**Published:** 2015-10-14

**Authors:** Cordelia Mühlenbeck, Katja Liebal, Carla Pritsch, Thomas Jacobsen

**Affiliations:** 1 Department of Education and Psychology, Evolutionary Psychology, Freie Universität Berlin, Berlin, Germany; 2 Department of Education and Psychology, Comparative Developmental Psychology, Freie Universität Berlin, Berlin, Germany; 3 Graduate School “Languages of Emotion”, Freie Universität Berlin, Berlin, Germany; 4 Experimental Psychology Unit, Helmut Schmidt University—University of the Federal Armed Forces Hamburg, Hamburg, Germany; University of Salamanca- Institute for Neuroscience of Castille and Leon and Medical School, SPAIN

## Abstract

Research on colour preferences in humans and non-human primates suggests similar patterns of biases for and avoidance of specific colours, indicating that these colours are connected to a psychological reaction. Similarly, in the acoustic domain, approach reactions to consonant sounds (considered as positive) and avoidance reactions to dissonant sounds (considered as negative) have been found in human adults and children, and it has been demonstrated that non-human primates are able to discriminate between consonant and dissonant sounds. Yet it remains unclear whether the visual and acoustic approach–avoidance patterns remain consistent when both types of stimuli are combined, how they relate to and influence each other, and whether these are similar for humans and other primates. Therefore, to investigate whether gaze duration biases for colours are similar across primates and whether reactions to consonant and dissonant sounds cumulate with reactions to specific colours, we conducted an eye-tracking study in which we compared humans with one species of great apes, the orangutans. We presented four different colours either in isolation or in combination with consonant and dissonant sounds. We hypothesised that the viewing time for specific colours should be influenced by dissonant sounds and that previously existing avoidance behaviours with regard to colours should be intensified, reflecting their association with negative acoustic information. The results showed that the humans had constant gaze durations which were independent of the auditory stimulus, with a clear avoidance of yellow. In contrast, the orangutans did not show any clear gaze duration bias or avoidance of colours, and they were also not influenced by the auditory stimuli. In conclusion, our findings only partially support the previously identified pattern of biases for and avoidance of specific colours in humans and do not confirm such a pattern for orangutans.

## Introduction

The capacity for trichromatic colour vision has evolved in many primates, including humans. Catarrhines (Old World monkeys and apes) are routine trichromats [1, 2], while most platyrrhines and some strepsirrhines also have a capacity for trichromatic colour vision in that some individuals are trichromats (polymorphic trichromats) [3–6]. Two hypothesized functions for the evolution of trichromatic colour vision are that this capability provided advantages in detecting ripe fruits and in detecting young leaves [2, 7]. In this context, it is assumed to have provided advantages for diurnal species [5]. However, there does not seem to be a consistent difference between fruit predominantly consumed by dichromats and that predominantly consumed by trichromats [2, 8]. Another hypothesized function of trichromatic colour vision is the advantage of being able to discriminate modulations in the skin colour of conspecifics for extracting information about emotional states, socio-sexual signals and threat displays [9]. In addition, functions of colour preferences have been widely discussed in relation to mate choice, both in humans and other primates [10–12], and also in relation to crypsis, communication and physiological functions [10]. Furthermore, as Humphrey [13] pointed out, “signal colours commonly have three functions: they catch attention, they transmit information and they directly affect the emotions of the viewer”. Hence, sensitivity to signal colours probably also influences preferences for specific colours. This raises the question, in regard to humans and non-human great apes, whether shared colour biases could have evolved together with the evolution of colour vision. Moreover, Osorio and Vorobyev [8] noted that there are different perspectives regarding the evolution of sensory systems: “One is that communication signals evolve in response to a fixed sensory system [14], the other is that senses and signals co-evolve as a specialised communication system.” If senses and signals co-evolved, it could be that biases for specific colours are linked to this communication system.

Various studies have examined colour preferences in primates other than great apes, mostly in relation to sexual contexts [15–20], but also in relation to object preferences [21]. Studies on the colour preferences of non-human great apes which are not related to mate information but rather to food and object choice have shown that zoo-housed orangutans (*Pongo pygmaeus*) prefer coloured to non-coloured monkey chow [22] and that gorillas (*Gorilla gorilla gorilla*) and chimpanzees (*Pan troglodytes*) both have a preference for blue and green objects over red ones [23]. Wells et al. [23] therefore concluded that the colour preferences of gorillas and chimpanzees resemble those of humans, suggesting that for the three species, there is a common innate relationship between colours and behavioural reactions, that is, the approach or avoidance behaviour that is provoked. However, the use of specific contexts to study colour preferences (such as reproductive contexts or contexts involving objects or food) gives rise to biases, because each species has adapted differently to different contexts. Hence, in comparisons between different species, the correct analysis of context-related preferences may be difficult. Studies that focus on species comparisons should therefore use colour stimuli that are not related to a specific context.

The extensive body of research on colour preferences in humans (for a review, see [24]) has found that blue is always most preferred and yellow is always least preferred [25–29]. The findings differ only in regard to preferences for the colours purple, red and green. Furthermore, research on the influence of colours on human emotions has suggested that a participant’s age [30] as well as saturation and brightness of the colour [31] play an important role in the association of a colour with an emotion. Brighter colours are judged more pleasant, and saturated, darker colours are judged more arousing, while hue plays only a minor role in the influence on mood [24]. However, despite the extensive body of research on human colour preferences, there is no consistent pattern in the conclusions which have been drawn from findings about the association between emotions and colours. Aesthetic perceptions are guided by different overlapping influences, such as person–situation interactions, the domain in which a specific colour occurs [32] and the interaction of biological factors with social processes, and as a result researchers give rather inconsistent reasons for human colour biases. Similarly, and relevant for the present comparative study, primate colour signals also occur in different contexts, such as mate choice (reproductive context) and threat displays (competitive context).

In the acoustic domain, a variety of experimental studies have also been conducted on the perception of consonant and dissonant sounds and their influence on human emotions (for reviews, see [33] and [34]). Functional imaging studies have suggested that consonant and dissonant sounds activate the same brain regions as other pleasant and unpleasant stimuli, respectively [35, 36]. Moreover, consonant sequences of sounds are perceived as pleasant and dissonant sequences as unpleasant when heard in isolation, but dissonant sequences can disperse and become harmonious and pleasant when they are integrated in a composition [37]. The few studies that have investigated the perception of consonant and dissonant sounds across cultures [38, 39] have found similar perceptions in humans regardless of their cultural background, suggesting that human sound biases in terms of being pleasant or unpleasant are most likely innate. This is supported by developmental studies conducted by Zentner and Kagan [40, 41] and Trainor et al. [42], who found that 4-month-old infants already preferred consonant over dissonant sounds—which led McDermott and Hauser to conclude that the preference for consonant sounds is innate and emerges independently of experience [34]. If the preference for consonant sounds is innate in humans, it most likely evolved in the primate lineage and should thus be shared by other primates. However, apart from another study by McDermott and Hauser [43], which did not find similar approach–avoidance reactions to consonant or dissonant sounds in tamarins (a species of New World monkeys), virtually nothing is known about the biases of non-human primates in the auditory domain regarding sounds which are detached from vocal information. Thus, it remains an open question whether great apes, which are more closely related to humans than are New World monkeys, share the bias for consonant sounds with humans.

Furthermore, if colours provoke a psychological reaction (bias for or against) or a behavioural reaction (approach or avoidance), the question arises whether a consonant or dissonant sound can amplify the reactions associated with specific colours. Multimodal signalling has been examined in many studies in an evolutionary context [44, 45]. One of the functions of multimodal signalling could be that the psychological effects of visual cues can be enhanced by non-visual cues (for a review, see [45]). Multimodal signalling occurs in very different combinations of modal domains, such as coloration and odours, but also gestures and vocalizations [46–48]. As most primate vocalizations are species-specific, we needed to find a different acoustic stimulus that could be used across species and that was also known to provoke approach–avoidance reactions. Therefore, we used consonant and dissonant sounds and conducted a non-invasive comparative eye-tracking study of orangutans—a species of non-human great apes—and humans, to test the duration of their fixations on different colours with and without simultaneous auditory stimuli.

The non-invasive eye-tracking technique has been successfully applied by Kano and colleagues [49–51] to investigate the gazing patterns of great apes, including orangutans. These previous studies mostly focused on how great apes process visual information such as facial and bodily expressions when they look at pictures of conspecifics or humans. In contrast to these studies, we used colours, which are also visual stimuli, but without any relation to objects or persons. Despite the complexity that arises in the domains of both colour and sound biases due to the influence of human participants’ cultural and personal backgrounds, and also despite humans being more familiar with certain colours than orangutans, we combined a basic visual stimulus with a basic auditory stimulus because we needed stimuli that could be used across different primate species, free of any biases that might be caused by the colours being combined with objects or other types of stimuli. Thus, although presenting colours and sounds detached from any related information, such as mate information or objects (for colours) and vocalizations (for sounds), could be viewed as having low ecological validity, it reduces the biases associated with such related information.

Our aim was to investigate 1) whether there is a similar pattern of gaze duration biases and avoidances related to different colours in the two species and 2) whether humans and orangutans associate specific colours with auditory information—specifically, whether the approach or avoidance of a colour cumulates with the approach or avoidance of an auditory stimulus. We examined gaze fixation durations for the four colours blue, red, yellow and green, both separately and in combination with an auditory stimulus comprising a consonant or dissonant triad and chord (a triad is a set of three tones played one after another and the chord was the same set of three tones played simultaneously, directly after the triad). If colour biases are innate, they should be similar across humans. If they are shared with other species and thus represent an evolutionarily older trait, the gaze duration biases for specific colours should be similar in humans and orangutans. The gaze duration biases in combination with auditory information should also be similar.

## Methods

### Participants and Ethics Statement

We tested 15 German adults (mean age 30.3; age range 20–47 years; 6 male, 9 female) at the Freie Universität Berlin and 8 Sumatran orangutans (*Pongo abelii*; mean age 14.5; age range 3–32 years; 3 male, 5 female) at the Wolfgang Köhler Primate Research Centre (WKPRC) of the Max Planck Institute for Evolutionary Anthropology (MPI-EVA) in Leipzig, Germany. For the human participants, there was no need for further approval by an ethics committee, since the research and testing procedure were in accordance with the ethical recommendations of the Deutsche Gesellschaft für Psychologie (DGPs; German Psychological Association). The participants were students at the Freie Universität Berlin and they voluntarily participated in the study. Before testing, we obtained their written informed consent.

The orangutans were all members of a single group consisting of 11 individuals at the WKPRC who lived in semi-natural indoor (230 m^2^) and outdoor (1680 m^2^) enclosures with regular feedings, daily enrichment and water ad lib. The research at the WKPRC was conducted in accordance with the recommendations of the Weatherall report [52]. All orangutans participated in the study voluntarily, were able to stop participating at any time, and were never food- or water-deprived. Research was conducted in the observation rooms (25 m^2^). No medical, toxicological or neurobiological research of any kind is conducted at the WKPRC. The research was non-invasive and strictly adhered to Germany’s legal requirements. The study was ethically approved by an internal committee at the Max Planck Institute for Evolutionary Anthropology and was carried out in strict accordance with the guidelines of EAZA [53], WAZA [54] and ASAB [55].

### Apparatus and stimuli

For both groups, we used a screen-based Tobii T60 eye tracker (60 Hz, Tobii Technology) with an infrared corneal reflection technique, integrated in a 17-in TFT monitor (screen resolution 1280 × 1024 pixels) operated via an external laptop with Tobii software (version 2.1). Such external eye trackers allow the participants to move freely and therefore enable measurement of more natural viewing behaviour. A plexiglass panel separated the eye tracker from the orangutans and enabled the accurate measurement of their eye movements. A flexible tube was run through a small hole in the panel to connect to a bottle of diluted grape juice. This served to place the orangutans in front of the eye tracker at a distance between 60 and 70 cm, since they could drink while looking at the screen. For both humans and orangutans, the lighting and background sound conditions were kept similar and constant to control for their influence on the gazing behaviour of the participants.

The study consisted of three experimental conditions, the colour-only condition, the colour–sound condition, and an additional sound-only condition. The colour-only condition tested biases for one of the four colours red, green, yellow and blue. The colour–sound condition combined the colour-only condition with different consonant or dissonant major triads and chords. The purpose of the sound-only condition was to measure the participants’ pupil dilation when hearing the consonant and dissonant sounds; however, because the orangutans rarely gazed at the screen in this condition, the data set included too many data gaps and was not considered for further analysis. Therefore, we only report the colour-only condition and the colour–sound condition. Regarding the sound-only condition, descriptive statistics for both groups and inference statistics for the human sub-sample are provided as supporting material.

The two types of dissonant triads and chords were an augmented E (dissonant 1) and a diminished D-sharp (dissonant 2) triad plus chord. The two types of consonant triads and chords were a minor E (consonant 1) and a minor D (consonant 2) triad plus chord. Each colour stimulus consisted of a combination of two different colours. These were created by combining each colour with the remaining colours. The position of each colour was counterbalanced to appear once on the right and once on the left, so that altogether 12 colour stimuli were created (i.e., red-yellow, red-green, red-blue, blue-yellow, blue-green, blue-red, yellow-green, yellow-red, yellow-blue, green-yellow, green-blue, green-red). Each stimulus was 880 × 547 pixels. The blue had a hue of 230 degrees with a saturation of 100% and a brightness value of 50%; the yellow had a hue of 60 degrees with a saturation of 100% and a brightness value of 60%; the red had a hue of 360 degrees with a saturation of 100% and a brightness value of 100%; and the green had a hue of 125 degrees with a saturation of 100% and a brightness value of 70%. It should be noted that cone peak sensitivities and receptor densities vary across different species, and there are slight differences between humans and orangutans, although they are both trichromats. Visual models are available for comparing colour vision in different animals, such as those elaborated by Endler and Mielke [56] or Stoddard and Prum [57], but these models require knowing the cone peak sensitivities, receptor densities and cortical connectivity of both species, and these are not known in detail for orangutans. Hence, we relied on the similarities between the two species and used the same colours for both.

For the colour-only condition, two randomized sets were arranged, each containing the 12 colour stimuli. Each participant was presented with only one of the two randomizations. Each stimulus was presented for four seconds, followed by the presentation of a black cross in the middle of a white screen for one second to centre the participant’s gaze and avoid the possibility of a fixation on one stimulus influencing the next fixation. It was necessary to design the stimuli in colour pairs and not, for example, with single colours, in order to be able to analyze each participants’ gaze data for each colour in relation to their fixations on the other colours. The use of two colours in one stimulus means that the gaze of the participants can be attracted by two colours, which makes it possible for the participants to have longer fixations on one colour than on the other—whereas if they were presented with only one colour at a time, their mean fixation durations for all colours would be the same, because their gaze could not be attracted by different parts of the stimulus. Furthermore, through presenting all colours in both positions (left/right) and combined with all other colours, possible side biases or position biases (influence of the combination in which the colours are presented) were averaged out. The reason for using the mean fixation duration rather than the summed fixation durations stems from gaps in the data, which occurred due to the possibility of the eye tracker losing track of a participant’s eyes or a loss of attention on the part of the participants. The orangutans turned their heads or moved away from the eye tracker more frequently than the human participants. If we simply summed the fixation durations for each colour, a comparison between the colours would be meaningless. Due to the different number of data points, for example, we might be comparing the summed duration of five gazes fixated on yellow with the summed duration of only three gazes fixated on red, and the former is likely to be larger than the latter simply because of the different number of data points in the latter case. However, by using mean fixation durations, we avoided this problem and ended up with truly comparable information about the participants’ gazes at the different colours which allows us to infer biases and avoidance behaviours. According to Just and Carpenter’s eye–mind hypothesis [58, 59], there is a strong correlation between where one is looking and the most important point of attention; hence the mean duration of the gaze points gives us information about these biases or avoidance behaviours.

The total duration of the colour-only condition (for each randomization) was one minute. In the colour–sound condition, the 12 colour stimuli were presented together with the four different sound sequences in such a way that each colour combination was presented together once with each sound sequence. The triads plus chord had four beats spanning the four seconds of the presentation of one colour stimulus—three beats for the triad and one for the chord. When the black cross was presented, there was no auditory stimulus. The colour–sound stimuli were divided into four different parts, in randomized orders, so that each part was presented for a duration of one minute. Each participant had to watch each part, but the division of the colour–sound stimuli into parts was necessary because it allowed the different parts to be distributed over different testing days for the orangutan group. For the human group, the different parts of the colour–sound condition were presented on the same testing day.

### Testing Procedure

The orangutans had previously participated in eye-tracking studies and thus were used to looking at screens, while the humans had no eye-tracking experience but were used to looking at TV screens. Therefore, no training was necessary to accustom either group of participants to the experimental setting. Before testing, we made sure that the orangutans did not display any fear or stressful reactions in response to the unfamiliar sounds. The human participants merely received viewing instructions and were informed of the overall purpose of the study—to compare their visual perception to that of orangutans—without being told that the goal was to analyse colour preferences with the influence of sounds. To increase tracking accuracy, we conducted a manually changed two-point calibration for the orangutans and an automated five-point calibration for the humans to adjust the eye tracker to their eyes. The participants had to follow the points with their eyes while the eye tracker caught their corneal reflections and saved an eye model for each participant after testing the tracking accuracy. To reach a high spatial resolution for the eye tracking (Tobii standard: distance of 0.5 degrees between measured and intended gaze points), the calibration was repeated until it showed almost the same accuracy for all participants. The humans were tested in one session, including all conditions, with a total duration of approximately 6–7 minutes, depending on the time needed for calibration. The orangutans each had several sessions, since we presented only the colour-only condition or one part of the colour–sound condition per day. This was necessary to keep the orangutans interested in participating in the study, because they had a shorter attention span than the human participants. During the testing, the apes received food rewards to hold their attention. The reward was given to them only for participating in the experiments, but never for their gaze behaviour. Because of the orangutans’ shorter attention span, the recordings had missing data when the orangutans moved away from the eye tracker or turned their heads. We filled the data gaps by repeated measurements of the same entire trial, to ensure that we obtained recordings for almost the same number of stimuli for the orangutans as for the human participants. To make sure that these repeated measurements did not result in more data (duplicate recordings) for the orangutan group, we filtered out duplicate recordings of the same stimuli, as described in the next section on data analysis. No measurements needed to be repeated for the humans. For the orangutans, an entire testing session lasted approximately 10 minutes per day, including the calibration and any repeated measurements.

### Data analysis

We defined fixations as instances in which the eye gaze remained within a radius of 50 pixels for longer than 100 ms. The angular position of the eyes was recorded with a frequency of 60 Hz and matched to the coordinate system of the stimuli on the monitor. No corrections of the raw tracking data were conducted. As mentioned above, measurements had to be repeated in the orangutan group. Because the entire trial was presented again, the data gaps could be filled, but duplicate recordings for the same stimuli could also occur. From these duplicate recordings, the first recording which displayed a viewing pattern of a minimum of two gaze points was used for analysis. This ensured that for the orangutans, only the visual processing of unknown objects was analyzed, in order to be comparable to the viewing of the human participants, who had seen each stimulus only once. The later duplicate recordings were not used for the analysis because they represented the visual processing of an already known stimulus.

We log transformed the dependent variable (*mean fixation duration*) to achieve better interpretability and more symmetrically distributed data. The distribution asymmetry in the data for the mean duration of the single gaze points was similar in both groups.

### Statistical methods

To test whether the mean duration of fixations on the stimuli was influenced by the colours (factor *Colour*) or the species-specific differences (factor *Group*), we generated general linear mixed models [60, 61] of increasing complexity, starting with the simplest model comprising the *mean fixation duration* as the dependent variable, the factor *Group* as a fixed effect and *Subject* as random effects (model 1). To test the effects of the two variables *Sex* and *Age*, we included the factor *Sex* in model 1 to form model 2. To test for the effect of *Sex*, we tested the significance of both models, model 1 and model 2, using a likelihood ratio test (using the R function ‘anova’ with the argument ‘test’ set to “Chisq”), fitted with maximum likelihood [61]. We then included the factor *Age* in model 1 to form model 3. To test whether *Age* had an effect on our dependent variable, we again ran a likelihood ratio test on model 1 and model 3. We continued this procedure and included the second main factor, *Colour*, in model 3 to form model 4, which therefore comprised *mean fixation duration* as the dependent variable, *Group* and *Colour* as fixed effects and *Subject* as random effects. We ran the same likelihood ratio test as described above to test for any effects of the factor *Colour*. We then included a cross-level interaction [62] between *Colour* and *Group* to form model 5. We thereby tested for any species-specific differences in the factor *Colour*.

Next, we included the factor *Sound* in model 5 to form model 6, to test for any effect of the factor *Sound* on the mean fixation duration. We then tested for an effect of *Sound* on the effect of *Colour* by including a cross-level interaction of *Colour* and *Sound* to form model 7, which now comprised the cross-level interactions Colour*Group and Colour*Sound as fixed effects and *Subject* as random effects. Finally, we replaced the cross-level interaction Colour*Sound by the cross-level interaction Group*Sound to test for any species-specific effects of *Sound*, resulting in model 8. Model 8 was an extension of model 6 and hence, it was also compared to model 6.

We had controlled for a possible side (left or right) bias of the participants by presenting all colours on both sides and thus balancing such a bias. Correlations between the fixed effects were not assumed. We checked whether the assumptions of normally distributed and homogeneous residuals were fulfilled by visually inspecting a qq-plot and the residuals plotted against fitted values (both indicated no obvious deviations from these assumptions). The model stability was examined using the function ‘influence’ from the R-package influence.ME [63]. Inspection of *df*-betas, Cook’s distance and the sigtest revealed that some subjects had an influence according to classical cut-off criteria, but according to content-based criteria these were classified as not excludable. Variance inflation factors (VIFs [64]) were derived using the function ‘vif’ from the R-package car [65] applied to a standard linear model excluding the random effects, and these indicated that collinearity was not an issue. All models were fitted in R [66] using the function ‘lmer’ from the R-package lme4 [67].

## Results

The likelihood ratio test between model 1 and model 2 showed no effect of *Sex*, and the likelihood ratio test between model 1 and model 3 showed no effect of *Age*. As *Sex* and *Age* showed no effect, they were excluded from the subsequent analyses. The likelihood ratio test between model 3 and model 4 indicated that including the factor *Colour* in model 4 improved the fit of the model to the data, but not significantly (*p*-value = 0.062; Table 1). The likelihood ratio test between model 4 and model 5 indicated that including the cross-level interaction between *Colour* and *Group* in model 5 significantly improved the fit of the model to the data (*p*-value = 0.015), which means that species-specific differences existed in relation to the factor *Colour*. Table 1 shows the results of the likelihood ratio tests for all generated models for colours only.

**Table 1:** Likelihood-ratio tests of model comparisons for colours only. The table shows the results of the model comparisons for colours only. All models had *mean fixation duration* as the dependent variable and *Subject* as random effects.

Model	included fixed effects	model comparison	values for models		values for model comparisons		
			*df*	AIC	Chi sq.	*df*	*p*-value
model 1	*Group*		4	99.708			
model 2	*Group* + *Sex*	compared to model 1	5	101.668	0.0408	1	0.840
model 3	*Group* + *Age*	compared to model 1	5	101.565	0.1432	1	0.705
model 4	*Colour* + *Group*	compared to model 3	7	102.06	3.4797	1	0.062
model 5	*Colour* * *Group*	compared to model 4	10	97.617	10.446	3	0.015

The likelihood ratio test for the comparison of models 5 and 6 found no effect of *Sound*. The likelihood ratio test for models 6 and 7 showed no effect of *Sound* on the effect of *Colour* (cross-level interaction between *Colour* and *Sound)*. The likelihood ratio test of the comparison between models 6 and 8 showed no species-specific effects of *Sound*. Table 2 shows the results of the likelihood ratio tests for all models generated to test the effects of *Sound*.

**Table 2:** Likelihood-ratio tests of model comparisons for colours and sounds. The table shows the results of the model comparisons for colours and sound. All models had *mean fixation duration* as the dependent variable and *Subject* as random effects. Model 4 and model 5 were built like the models 4 and 5 for colours only (Table 1). Models 7 and 8 were both extensions of model 6 and hence were both compared to model 6.

Model	Included fixed effects	model comparison	values for models		values for model comparisons		
			*df*	AIC	Chi sq.	*df*	*p*-value
model 4	*Colour + Group*		7	480.43			
model 5	Colour * Group	compared to model 4	10	476.81	9.6196	3	0.022
model 6	Sound + Colour * Group	compared to model 5	13	479.32	3.4862	3	0.323
model 7	Colour * Group + Colour * Sound	compared to model 6	22	492.70	4.6201	9	0.866
model 8	Colour * Group + Group * Sound	compared to model 6	16	483.39	1.9323	3	0.587

**Fig 1:**
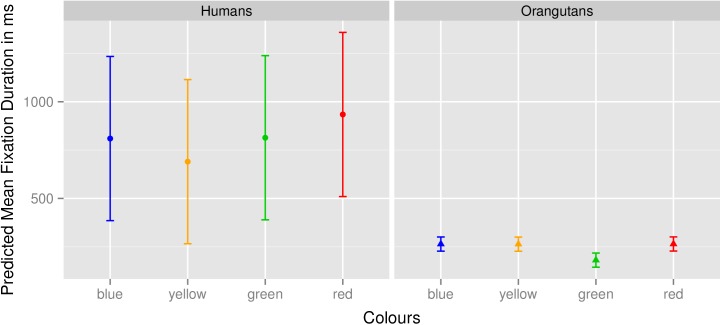
Predicted mean fixation duration in ms for the four colours. Fig 1 represents the predicted mean fixation duration in the two groups for the four colours. Values were calculated with a non-log-transformed dependent variable and represent the fixation duration in milliseconds. The left box shows the values for the human participants, the right box those for the orangutans.

**Fig 2:**
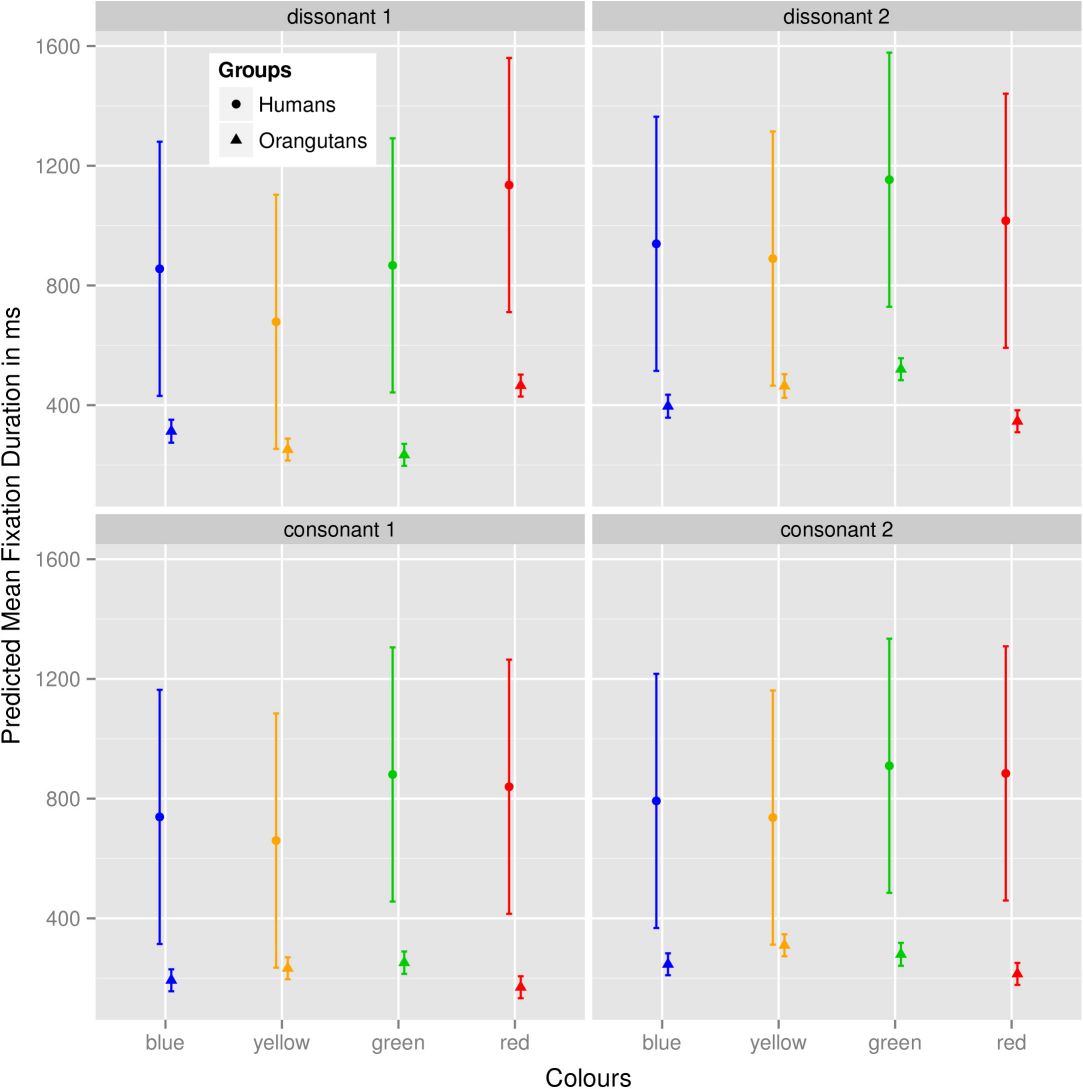
Predicted mean fixation duration in ms for the four colours and sounds. Fig 2 represents the predicted mean fixation duration in the two groups for the four colours together with the four sounds. Values were calculated with a non-log-transformed dependent variable and represent the fixation duration in milliseconds. The four boxes represent the four types of sound, and the two groups are represented by the different shapes of the dots.

Since *Sound* showed no effects (see Table 2), model 5 (with *mean fixation duration* as the dependent variable, the cross-level interaction of the factors *Colour* and *Group* as fixed effects and *Subject* as random effects) was the only model that showed a significant effect. Model 5 revealed that species-specific differences arose in the *mean fixation duration* for the four colours, in that the human group observed yellow for significantly shorter durations than the colours red (Estimate: 0.257; *t*-value: 3.36; *p* = 0.001) and green (Estimate: 0.244; *t*-value: 3.20; *p* = 0.001). For the colour blue, again in the human group, there was no visible effect indicating that it was observed for significantly longer durations than yellow (blue: Estimate: 0.140; *t*-value: 1.83; *p* = 0.068). Using the Šidák correction, the critical *p-*value for the tests of the regression weights is 0.007. We calculated the *p*-values from the *t*-values of the colours red, green and blue with respect to yellow in the intercept, with *df* for level-1 regression, since *Colour* is a characteristic of the stimulus and not of the subject. This works for models with fewer than 40 level-2 entities ([68], p. 95). (A detailed list of all calculated Estimates, *t*-values and *p*-values for all colours can be found in S2 Table). In the orangutan group, there were no such effects visible in the mean fixation duration, which indicates that the humans avoided yellow (and as a trend also blue, as blue was not observed significantly differently than yellow), while the orangutans did not (please see also Fig 1). When combined with the four sounds, the fixation durations remained almost the same as they were for the colour-only condition (please see Table 2 and also Fig 2). Fig 1 shows the mean fixation duration for the four colours in the two groups for the colour-only condition, here represented with the non-log-transformed dependent variable. As described above, it is clear that yellow was observed for shorter durations than the other colours in the human group, but not in the orangutan group. Fig 2 shows the mean fixation durations in the two groups for the four colours together with the different sounds. The mean fixation duration remained almost the same in both groups, which indicates that the sounds did not have a significant influence on the fixation duration.

## Discussion

Our finding that humans avoided yellow confirms findings of the previous studies with humans [25–29] mentioned in the introduction. However, in contrast to these studies, we did not find evidence for a clear bias for blue in humans, because the mean fixation durations for yellow vs. blue, and also for blue vs. green and blue vs. red, did not differ significantly. This can be seen in the fact that the Estimates for red (Estimate: 0.257; *t*-value: 3.36; *p* = 0.001) and green (Estimate: 0.244; *t*-value: 3.20; *p* = 0.001) are very similar, and blue lies between those values for yellow and red/green. The orangutans did not show any bias for or avoidance of specific colours, which seems to support a study by Barbiers [22], who also found no overall preference for a specific colour. In Barbiers’ study, when orangutans were confronted with differently coloured food items (monkey chow in red, blue, green and orange), only one of the five orangutans showed a clear preference for red monkey chow [22]. This also seems to indicate that orangutans are different from gorillas and chimpanzees, since Wells et al. [23] concluded that these species, which are more closely related to humans, show a colour preference for blue and green, similar to humans. However, based on these very few studies with non-human great apes, it is not yet possible to determine whether this is evidence for a difference between humans, chimpanzees and gorillas on the one hand and the more distantly related orangutans on the other, or whether the different findings of the present study and of Wells et al. [23] are due to differences in the methodology used. Still, the differences suggest that the capacity for trichromatic colour vision did not evolve together with a shared bias for specific colours. The advantages of trichromatic colour vision discussed in the studies described in the introduction to this paper were advantages for detecting ripe fruits and young leaves [2, 7] and for discriminating skin colour modulations [9]. The present study’s findings neither support nor disagree with the suggested reasons for the evolution of trichromatic colour vision, because a clear shared bias for one colour was not found in the two groups and hence cannot be used to speculate about a shared evolutionary reason for trichromatic colour vision. The results of this study show differences between humans’ and orangutans’ relative gaze fixation durations when confronted with these colours, which suggests that the two groups react differently to the colours when confronted with them.

Regarding the combination of colours with an auditory stimulus, we found no influence of either consonant or dissonant sounds on colour biases. However, contrary to our predictions, human avoidance of the colour yellow was not modified when this colour was combined with sound. Since previous studies have found that isolated dissonant sounds provoke an avoidance reaction [37], we expected that this reaction would cumulate with an avoidance reaction for a specific colour. The avoidance of dissonant sounds has been supported by different studies, including comparative studies which have investigated the perception of consonant and dissonant chords in non-human primates (for a review, see [69]). For example, rhesus monkeys are able to clearly discriminate between consonant and dissonant chords, with activation of similar brain regions to those in humans [34]. However, it remains an open question whether the monkeys’ activated brain regions and the ability to discriminate between consonant and dissonant sounds reflect the same pleasurable or aversive reactions found in humans [34]. At the age of 16 weeks, human infants already show these reactions and will turn away from dissonant sounds, even crying, but will turn towards consonant sounds, often smiling [40, 41, 70]. Our study’s finding of the absence of a cumulative effect from avoidance of dissonant sounds therefore suggests that perceptions of visual stimuli are not influenced by auditory stimuli.

While several studies [23, 24] have suggested similarities in colour preferences across different species and cultures, other studies have proposed, with regard to cross-cultural variations in colour meaning, that any effects of colour upon behaviour are learned [71, 72]. Thus, studies investigating the perception of colours and their representation in cultural usages and the corresponding languages have shown that across cultures, the meanings of colours are not independent of the syntax and semantics with which the colour names are used [72]. In this regard, the mere-exposure effect plays an important role: based on their degree of familiarity, spectators develop different attitudes towards observed objects in general and, more specifically, towards colours. This means that the contexts in which different colours have been used influence spectators’ attitudes and hence the approach or avoidance reactions they exhibited. This is also supported by Whitfield’s observation that “colours vary in regard to the extent in which they correspond with the learned specifications of different object categories” [73]. Similarly, Davidoff et al.’s [74] findings did not support the idea that colour categories are universal, though they admitted that neurons are selective toward wavelength, brightness and colour constancy, as discovered in monkeys [75, 76]. They concluded that a cultural group’s environment and language influence colour categorization. The differences in colour categorizations among languages show that language, culture and colour perception are interlinked. In addition to environment and language, there are other influences on individuals’ colour biases, such as world knowledge, education, historical change and individual- and group-specific leitmotifs, as Jacobsen [77] made clear. However, Crozier [24] and Adams and Osgood [25] conclude that despite obvious cultural differences, there are also many similarities between cultures in regard to the usage and meanings of specific colours. For example, the association of red with fire or blood is widely shared across cultures. But the importance of the context in which colours occur applies not only to humans, but also to other primates, as noted in the introduction. For example, red as a signal can be linked to a reproductive context, like the red swellings in female baboons [78], but also to a competitive context for social status among males, as in the scrotal colour of adult vervet monkeys [79]. Because of these different types of biological information that can be linked to colours, we conducted our study with colour stimuli that were designed to have a most basic form. Our stimuli contain the risk of low ecological validity, but they hold the advantage of minimizing any asymmetry in possible biases.

Since we did not find a shared colour bias between the two species, we cannot, in particular, support the idea that the association of red with a potential hazard (such as fire or blood; see above) has a biological root, as suggested by the different cross-cultural and primate studies mentioned above. We did not find any evidence for an avoidance of—or bias for—the colour red in humans and orangutans. We only found a clear avoidance of yellow in the human group, while red and green were fixated upon for equal durations. In addition, we cannot support previous findings that blue is the most preferred colour for humans [24, 25], as humans observed it only slightly longer than yellow in our study. Regarding the avoidance of the colour yellow, we can only speculate why humans but not orangutans avoid this colour. Since several cross-cultural studies covering a wide age range across childhood and adolescence [80–83] have found that the avoidance of yellow is present in both children and adolescents independent of their cultural background, a possible explanation which has been offered for this avoidance reaction is based on the perceived hue and brightness of the colours. Katz and Breed reported that “there was a distinct rise in the preference values of colours of short wave length and a corresponding decline in the values of colours of long wave length, as the children advanced in age” ([83], p. 255). While infants younger than three months preferred colours with longer wavelengths (red and yellow), after the age of three months [84] infants showed a preference for colours with shorter wavelengths (blue and green). However, why the preference for shorter wavelengths evolved and why it only emerges later in ontogeny is not clear. To summarize, on the one hand, approach or avoidance reactions to specific colours are strongly influenced by different cultural, group-specific and individual characteristics, as argued above; but on the other hand, studies with infants have seemed to consistently show that prior to cultural learning, there are approach reactions to specific colours. Finally, regarding cultural influences, one would expect that individual differences in colour approach or avoidance reactions would be stronger, which was not the case in our study.

## Conclusions

Our findings confirm previous findings that humans avoid yellow, but do not provide any evidence confirming a clear bias for blue. Unlike humans, orangutans showed neither biases for nor avoidance of specific colours. Hence, we conclude that the evolution of trichromatic colour vision does not eventually have to be accompanied by biases for specific colours. Furthermore, when we combined colours with different consonant or dissonant auditory stimuli, we found that the latter had no influence on the approach or avoidance reactions to the visual stimuli and hence conclude, within the restricted parameters of this study, that the bias for or avoidance of a specific colour seems to be independent of the perception of an auditory stimulus.

## Supporting Information

S1 Table General linear mixed model for mean pupil diameter for the human group. The table shows the results of the model for the mean pupil diameter (dependent variable) with *Baseline C*, *Sounds* and an interaction between the two as fixed effects, and *Subject* as random effects. Results are only given for the human group, as the data for the orangutan group displayed too many data gaps.
(XLS)

S2 Table Estimates, *t*-values and *p*-values for all colours and both groups, calculated from model 5. (XLS)

S1 Fig Mean pupil diameter in mm for baseline C and the four sounds for both groups. In the sound-only condition, a white screen with two parallel lines was presented on the eye tracker while a baseline (tone C) and one of the four different sounds (dissonant 1, dissonant 2, consonant 1, consonant 2; always a combination of a triad and chord) was played. This combination of baseline and sound was followed by a black fixation cross with no sound played and then by the next baseline–sound combination. The order of the baseline–sound combinations was randomized. With the eye tracker we measured the pupil diameter, and we compared the baseline and the triad–chord combination.
(EPS)

S1 Data Datasets for all three conditions and all participants. (ZIP)
